# Hypertension and diabetes patients’ perspective of challenges and their coping mechanisms in Mukono and Buikwe districts in Uganda – a qualitative study

**DOI:** 10.12688/openreseurope.13286.2

**Published:** 2023-09-22

**Authors:** Rawlance Ndejjo, Paineto Masengere, Fred Nuwaha, Isaac Ddumba, Hilde Bastiaens, Rhoda K. Wanyenze, Geofrey Musinguzi

**Affiliations:** 1 Disease Control and Environmental Health, School of Public Health, College of Health Sciences, Makerere University, P.O Box 7072, Kampala, Uganda; 2 Department of Health, Mukono District Local Government, Mukono, Uganda; 3 Department of Family Medicine and Population Health, University of Antwerp, Antwerp, Belgium

**Keywords:** challenges, coping mechanisms, diabetes, hypertension, lifestyle, self-monitoring, Uganda

## Abstract

**Background**: In sub-Saharan Africa, the burden of non-communicable diseases is steadily rising amidst a high prevalence of communicable diseases stretching the healthcare system. This study explored hypertension and diabetes patients’ perspective of challenges and their coping mechanisms in Mukono and Buikwe districts in Uganda.

**Methods**: This descriptive qualitative study involved four focus group discussions with 26 patients at four selected health facilities. All interviews were audio recorded, transcribed verbatim and data analysed following the thematic content analysis guided by the semantic approach with the aid of Atlas ti 6.0.15 software.

**Results**: Five themes were identified regarding challenges and coping mechanisms of patients in managing their conditions. 1) Inadequate opportunities for diagnosis, with community screening supporting identification of patients. 2) Accessing care came amidst transport challenges, absence of health workers and the lack of essential supplies for monitoring conditions. Patients borrowed transport funds or trekked to health facilities and some formed groups to contribute resources to buy equipment and supplies. 3) Access to medications was affected by frequent drug stockouts at public health facilities which pushed patients to purchase own drugs or obtain these through friends and networks. However, other patients resorted to cheaper herbal remedies. 4) Monitoring and managing conditions was affected by insufficient knowledge and opportunities for self-monitoring. Information from health workers and experiences from peers bridged the knowledge gap while private facilities or community health workers supported self-monitoring. 5) Adopting changes in behaviour was challenging but patients fitted these within their usual routines and mobilised family members to also adopt lifestyle changes while ignoring those they deemed unrealistic.

**Conclusions**: The coping mechanisms patients adopted to manage their chronic conditions reflects self-care initiatives at the individual and community levels which could be reinforced and supplemented to better support and empower patients as steps are taken to address existing challenges.

## Abbreviations

CVD, cardiovascular disease; FGD, focus group discussion; NCDs, non-communicable disease; SPICES, Scaling up Packages of Interventions for Cardiovascular disease prevention in selected sites in Europe and Sub-Saharan Africa.

## Plain language summary

In many developing countries, many more people are now living with non-communicable diseases including diabetes and hypertension. However, since previously the health system was designed to manage infectious diseases such as malaria, tuberculosis and HIV, it is unprepared to deal with this emerging disease burden. In this study, we conducted interviews among hypertensive and diabetic patients to understand the challenges they faced in managing their conditions and how they coped in Mukono and Buikwe districts in Uganda. We found that hypertension and diabetes patients had inadequate opportunities to be diagnosed and community screening helped some obtain preliminary information that supported diagnosis. Patients also struggled to access health facilities due to transport challenges and sometimes unavailability of health workers and/or essential supplies, which they dealt with by borrowing transportation costs, walking to health facilities and organising themselves into a group to contribute resources to buy essential supplies. Frequent stockouts of medicines at health facilities also meant that patients had challenges accessing drugs and they thus bought their own drugs or resorted to the cheaper herbal remedies. Patients also struggled to monitor and manage their conditions and they used every opportunity to learn from each other or local community health workers. As patients struggled to adopt recommended lifestyle changes, they fitted these within their routines and recruited their family to adopt similar changes. From this study, we learn that patients are taking the initiative to manage their own conditions, which can contribute to further enhancing self-care interventions to better support them. Nevertheless, efforts are needed to address the challenges that patients face in managing hypertension and diabetes in Uganda and other developing countries.

## Introduction

Non-communicable diseases (NCDs) such as cardiovascular disease (CVD), diabetes, cancer, and lung disease are fast replacing infectious diseases in priority in sub-Saharan Africa (SSA). Hypertension was ranked in the third position among the leading causes of the burden of disease in low- and middle-income countries, contributing 4.2% among the risk factors for loss of healthy life ^1^. Between 2019 and 2045, it is estimated that the SSA region will have the greatest increase of 142.9% in the number of people with diabetes compared to elsewhere in the world ^2^. In Uganda, a national NCD risk factor survey reported the prevalence of hypertension among adults aged 18 to 64 years as 26.5% in 2014 ^3^. The prevalence of diabetes mellitus in Uganda varies, with up to 9% reported in some parts of the country, and continues to rise ^4– 6^. An increase in the incidence and prevalence of diabetes mellitus in children, adolescents and young adults has been attributed to rising levels of obesity, physical inactivity, and poor diets ^7, 8^.

The growing epidemic of chronic, non-communicable conditions in developing countries calls for prepared health systems. However, health systems in SSA countries were previously designed to manage infectious diseases, such as malaria, diarrhoea and respiratory infections and thus not prepared to deal with the rising burden of non-communicable conditions ^9– 12^. In Uganda, the situation is not any different with effective management of NCDs at health facilities hindered by underfunding, inadequate human resources and frequent stockouts of essential diagnostics and supplies ^13, 14^. A study on capacity of health facilities to manage hypertension in Mukono and Buikwe districts indicated challenges of unskilled staff, lack of guidelines, lack of equipment, drug stockouts and knowledge gaps ^14^.

Health systems challenges in managing NCDs cause the greatest brunt among chronic disease patients affecting their access to care and health-seeking behaviours ^15^. While a previous study in Mukono and Buikwe districts explored the factors influencing compliance and health-seeking behaviour for hypertension ^15^, the focus was at the community level. Moreover, most studies in SSA on the care and management of hypertension and diabetes have focused on socio-economic constraints concerning challenges at health facilities mainly providing the providers perspective ^11– 14, 16, 17^. More research on patient perspectives is thus needed to guide policies to improve primary care of patients with NCDs ^18^. The current study, conducted as part of the situational analysis for the Scaling-up Packages of Intervention for CVD prevention in selected sites in Europe and sub-Saharan Africa (SPICES) project to inform CVD prevention and control interventions ^19^, explored hypertension and diabetes patients’ perspective of challenges and their coping mechanisms in Mukono and Buikwe districts in Uganda.

## Methods

### Ethics approval and consent to participate

This study obtained ethical approval from the Higher Degrees Research and Ethics committee of Makerere University School of public Health (protocol 513) and was registered by the Uganda National Council for Science and Technology (HS 2477). Permission to conduct the study was sought from the district authorities and the health facilities. Patients provided written informed consent before participation in the study.

### Study area

This study was conducted in Mukono and Buikwe districts in Central Uganda within approximately 35.0 km and 57.4 km of Uganda’s capital, Kampala, respectively. The districts have a combined population of 1,000,000 persons of whom 70% reside in rural areas ^20^. The districts are served by health facilities at different levels ranging from: health centre II at the parish level providing an out-patient clinic, treating common diseases and offering antenatal care; health centre III at the sub-county level with a general outpatient clinic and a maternity ward; health centre IV at the county with wards for men, women, and children and in-patients services; and hospitals at the district level providing specialised clinics including for chronic disease as per the country’s organization of health services. In addition to the government health facilities, there are private facilities some for profit and others not for profit. The two districts provide a combination of rural, urban, and semi-urban Uganda ^20^ and previous research indicated a high prevalence of hypertension ^21^.

### Study design and population

This was a descriptive qualitative study that involved exploring experiences of managing hypertension and/or diabetes among patients at selected health centre IVs in Mukono district and hospitals in Buikwe district where they received their care from. These facilities were selected because they provided chronic disease services. These high-level facilities were selected because they had chronic care clinics where care for hypertension and diabetes was better structured and had a high client volume.

### Data collection

Data were collected using focus group discussions (FGDs), each with about six to eight participants convened at the selected health facilities and data collection took a period of two weeks. FGDs allowed collective discussion of any ideas, issues and solutions among peers and probing of any issues ^22^. The study participants were selected purposively ensuring a good mix of age (younger and older clients) and sex (male and female) guided by a health worker in charge of the clinic reviewed available records and called selected patients by phone to invite them to the health facility on the agreed date and time. The exclusion criteria were clients who did not feel well enough to participate in the discussion. All invited participants came for the discussion and the health worker did not attend. A pretested FGD guide ^23^ with general questions and probes developed from previous literature ^15, 24^ guided the discussions that were moderated by one researcher (RN) while another, both Public Health graduates and researchers with experience conducting qualitative research, took notes. The FGD guide was pretested among similar patients at two lower-level health facilities in the two districts. The FGDs lasted about an hour, conducted at the health facility in the most spoken language among the participants ( *Luganda*), was audio recorded and later transcribed verbatim in *Luganda* and translated into English. A total of four FGDs were conducted and supported a saturation of the study themes. There was no prior relationship between the researchers and study participants.

### Data analysis

A thematic analysis method following the semantic approach was used to analyse the study transcripts aided by Atlas ti 6.0.15 software (for which QDA Miner Lite data analysis software is a freely available alternative). The process involved reading of the transcripts several times by two researchers and generating an initial codebook (including aprior codes) highlighting challenges and coping mechanisms of patients which guided the data analysis. At the end of the coding process, similar codes were grouped to form sub-themes which fed into the study themes. Quotations have been used to supplement presentation of the study themes. Reporting for this study ^25^ followed the Consolidated Criteria for Reporting Qualitative (COREQ) Research guidelines ^26^.

## Results

### Characteristics of patients

A total of 26 patients participated in this study, 18 of whom had hypertension, three had diabetes and five had both conditions which they had had for a period of between one month and 28 years. Participants were aged between 34 to 74 years and most were female (19), married (16) and engaged in farming (14). Regarding education level, an equal number (12) had attained primary and secondary education as their highest level.

### Challenges and coping mechanisms among patients

There were five themes that highlighted the challenges and coping mechanisms of patients in managing their conditions. These were in: diagnosis with hypertension and diabetes, accessing care for conditions, accessing medications, monitoring and managing conditions, and adopting lifestyle recommendations as detailed below and summarised in Table 1.

**Table 1.** Summary of themes and sub-themes of challenges and coping mechanisms among hypertension and diabetes patients in Mukono and Buikwe districts in Uganda.

Themes	Sub theme – challenges	Sub theme - coping
Diagnosis with hypertension and diabetes	Inadequate opportunities for diagnosis	Taking advantage of any opportunities to screen
Accessing care for hypertension and diabetes	High transportation costs to reach health facilities	Borrowing transport funds from colleagues and sometimes trekking to facilities
	Absence of health workers from stations resulting in long waiting time	Keeping scheduled appointments and early morning queuing in order not to miss health workers
	Inadequate information provided by health workers and their lack of follow-up	Obtain information from friends, traditional practitioners and media, and sharing experiences with peers
	Inadequate screening facilities and supplies	Forming support group to mobilise resources to purchase basic equipment and supplies
Accessing medications	Frequent drug stockouts at public health facilities	Purchase of own drugs or access drugs through friends and social networks or skipping medications
	Expensive drugs at private-for-profit facilities	Obtain subsidized drugs from private-not-for-profit facilities or resorting to cheaper herbal remedies used alone or concurrently with conventional medicine
Monitoring and managing conditions	Inadequate knowledge about their conditions	Keenly obtain any information from health workers and sometimes peers to support self-management
	Few opportunities for self-monitoring conditions	Utilizing private facilities or community health workers for monitoring or purchasing required supplies through contributions by their patient’s groups
	Lack of reminders for taking medication affecting adherence	Peers and family members acting as reminders to take medication.
Adopting lifestyle recommendations	Changing behaviors and adopting lifestyle recommendations was challenging	Fitting lifestyle changes within usual routine and mobilizing family members to also adopt changes while ignoring ‘unrealistic’ recommendations

### Diagnosis with hypertension and diabetes

Patients shared what it took them to be diagnosed with hypertension or diabetes. The majority mentioned that they had symptoms that made them seek health care, contributing to diagnosis. Others highlighted that a visit from either a community health worker or researcher offered them an opportunity for screening for hypertension and referral to nearby health facility for diagnosis. Those who had symptoms expressed that it took a long time for them to be diagnosed.

“ *I have had high blood pressure for about 7 years now. I came to the hospital when both legs and hands were paralyzed. The doctor ordered for investigations after which I was diagnosed with high blood pressure.*” (Patient 7, FGD 1)

Late diagnosis was attributed to delays in seeking care among patients, the lack of screening for the disease at most health facilities or the lack of suspicion by health workers to request their blood pressure and/or blood sugar measurements. The patients thus took advantage of any available opportunities to screen and know their status including through community health workers within their villages.

“ *The community health workers came and measured my blood pressure and told me to go and seek help from the health facility. At the hospital, they found that my blood pressure was still high and I was given medication.*” (Patient 5, FGD 3)

Patients also noted that there were no proper systems for follow-up and referral to the next level of care.

### Accessing care for hypertension and diabetes

Due to the nature of their conditions, patients needed to access care from health facilities more frequently including to keep appointments for follow-up. However, the process came with challenges in transportation due to long distances to facilities and high transport costs. To cope, patients sometimes borrowed transport funds from their colleagues or trekked all the way to health facilities to keep their appointments. At the facilities, sometimes health workers were absent and patients had to wait for long hours to see them and thus many resorted to only visiting facilities on their clinic days when there is an assigned health worker or on scheduled appointment days. When they really needed to get to the health facilities on other days, patients reported early morning queuing in order not to miss health workers and be attended to earlier.

“ *Sometimes you wake up very early in the morning without breakfast so that you see the doctor and by midday you are still in the queue yawning. You can spend a full day and you have not seen the doctor nor received any medications; why not go to the pharmacy and buy some medication to relieve your pain?*” (Patient 1, FGD 4)

Another challenge in accessing care for patients was the health worker attitudes; although many were friendly and receptive, some were noted to be unsupportive in giving patients sufficient information to manage their conditions or address their many concerns. Patients thus reported reaching out to their peers and friends to fill their knowledge gaps. The other sources of information reported were media and traditional health practitioners.

“ *Some of us get medication advice from television and radio adverts and sometimes books on herbal remedies. When you go to the hospital, they check and prescribe medication, but you don’t have money to buy the drugs. Health workers give us knowledge which has been helpful, and we opt to use it.*” (Patient 6, FGD 4)

The other concern patients had was equipment at facilities not being functional or the lack of some supplies such as diabetes testing kits and not consistently monitoring their disease progression. Some patients thus mobilised themselves into groups that made frequent contributions to support purchase of supplies during their clinic days.

“ *We formed a patients’ association where we make a small contribution every time we come to the health facility and these funds support us to acquire a blood pressure machine or diabetes testing kits. The group also supports you when you have been admitted to a health facility by giving you some money.*” (Patient 3, FGD 2)

### Accessing medications

As chronic disease patients, they required a frequent supply of drugs for their conditions. Unfortunately, stock outs of drugs were common in the public health facilities, affecting reliability of accessing drugs and adherence to medications. Patients thus resorted to buying their own drugs where they could, and others looked to their social networks such as relatives working at health facilities to avail them essential drugs. Moreover, some patients reportedly shared drugs with peers as they looked for funds to buy their own. Other patients simply skipped medications until they got money, or drugs were availed at the health facilities.

“ *When you can’t buy the medicines and don’t have the money at the time, you have to just request from a friend until you get money to buy your own.*” (Patient 4, FGD 3)
“ *My friend who is a health worker gave me some medication to take which lasted for about a week. She told me that after I complete the medication, I should go back for a blood pressure checkup. She supports me in managing my condition.*” (Patient 4, FGD 4)

Overall, drugs were deemed expensive and thus patients found it very hard to sustain buying all essential drugs continuously from private-for-profit facilities. Some patients opted for the private-not-for-profit facilities where drugs were deemed cheaper despite some of these facilities being far away. In other cases, patients resorted to cheaper drug options such as herbal remedies alone or concurrently with conventional medicine.

“ *I started by buying drugs but couldn’t keep up because of their cost and so I decided to use herbal medicines. It has been one and a half years ever since and I still use them. I go to the health facility to monitor my blood pressure once in a while.*” (Patient 2, FGD 4)

### Monitoring and managing conditions

Patients also noted challenges in self-management and monitoring of their conditions. They noted that they lacked sufficient information regarding their conditions. They were thus keen to obtain any information from health workers and peers with whom they shared experiences on clinic days. Some diabetic patients also noted that they utilised every opportunity to learn how to self-inject to ease administering their treatments. In addition, patients noted that opportunities for self-monitoring were few, both at the public health facilities and in the community. Some patients thus reported visiting private facilities, for example to check their blood pressure, and others reached out to community health workers who had blood pressure machines.

“ *If they give us that medicine [insulin] and tell us the dosage and how to inject, we go ahead and self-inject as you won’t get a clinician to inject you very early in the morning and at night at 8:00pm. So we have to learn and practice.*” (Patient 6, FGD 2)

Another key aspect of self-management that patients were cognizant of was adherence to drugs and noted that they sometimes struggled to remind themselves to take their medication. They thus tried to keep a routine, and their peers and family members also reminded them to take medications and adhere to the scheduled time.

“ *You reach a time and you get fed up because taking drugs every day is not easy. Sometimes I get tired and tell my children to remind me. I always try to keep a routine and take the drugs before I go to bed and when I wake up in the morning.*” (Patient 3, FGD 3)

### Adopting lifestyle recommendations

Patients reported that due to their conditions, they had been told to make changes in their behaviours and lifestyles, which they reported to be challenging. Patients thus reportedly adopted changes in their lifestyle as a means to manage their conditions. These changes included having strict dietary regimens and engaging in physical exercises which they tried to fit within their usual routines as guided by health workers to which they found relief. Other patients stated that they encouraged their families to adopt similar lifestyles to offer them support.

“ *My doctor told me that I should reduce weight to about seventy-five kilograms and I was eighty-three then. So, I wake up early and walk so that I can shed off some kilograms though it was quite difficult at the start but I always do my best and wake up every day to walk. I am now 80 kilograms.*” (Patient 3, FGD 1)

On the other hand, patients found it hard to change previous habits and expressed that some of the lifestyle recommendations varied among health providers and others were unrealistic for their circumstances and so they found it very hard to keep up with some which they ignored. Moreover, some patients had other health conditions that limited their lifestyle choices, while others experienced pain due to the conditions or side effects from drugs.

“ *They stopped me from eating most foods and sometimes I cannot afford the foods I am required to eat. They stopped me from eating cassava and maize flour and most local foods. Now imagine in our rural life, how can you survive when they stop you from eating cassava and sweet potatoes and tell to only eat matooke [bananas] and potatoes? I really try but sometimes I fail and I am tempted to eat cassava. Any living person is supposed to eat.*” (Patient, FGD 1)

## Discussion

This study explored hypertensive and diabetic patients’ challenges and coping mechanisms in managing their conditions in Mukono and Buikwe districts in Uganda. The study found challenges and coping mechanisms along the following themes: diagnosis process, accessing care, accessing medications, self-monitoring and management, and adopting lifestyle recommendations.

Patients reported delays and few opportunities for diagnosis similar to previous studies in Uganda. These delays could be attributed to waiting for symptoms to appear or become severe ^24^, low knowledge levels ^27, 28^ and inadequate capacity of facilities to provide timely diagnosis for non-communicable disease conditions ^14^. Such delays in diagnosis of patients could increase the risk of developing complications ^29, 30^. The lack of diagnostics and unskilled personnel are some of the factors that have been reported to hinder early diagnosis of hypertension ^14^. In our study, access to care was also affected by health system factors such as absence of health workers from stations, long waiting time, poor health worker attitudes, faulty equipment, lack of follow-up by health facilities and drug stockouts. Other factors were the high cost of transportation to health facilities and expensive drugs. Similar barriers to healthcare access by patients with chronic illnesses have been reported elsewhere in developing countries ^11, 12, 16, 24, 28, 31– 34^.

We found that patients were eager to seek health care for their conditions although their efforts were continuously undermined by the existing health system challenges and their socio-economic conditions. Patients also reported personal challenges with adherence to medication and lifestyle recommendations. A systematic review of patient self-reported barriers of adherence to anti-hypertensive medication found factors such as remembering to take medication, patients’ beliefs about hypertension or antihypertensive medications, and patient self-efficacy to be influential ^35^. As recommended by many previous studies ^12, 18, 24, 28, 36, 37^, efforts are needed to address patient related barriers and health system challenges that affect access to and quality of care for chronic disease patients to lessen the burden in dealing with their conditions, increase their chances of adherence and quality of life.

Due to several challenges the patients faced, they devised strategies to cope. Visiting health facilities when they had symptoms and being screened by community health workers in their areas helped many to obtain a diagnosis. To access care, patients reportedly trekked to facilities when they lacked funds for transport to keep their appointments or went to the facilities earlier so that they are served first and avoid waiting long hours. Patients also formed groups to mobilise resources to support purchase of basic equipment and supplies. Moreover, patients frequently bought their own drugs or mobilised for these through their relatives, friends, and networks as these were frequently stocked out at facilities. Such coping mechanisms are a reflection of patients taking the lead to deal with their conditions and support one another and are in line with the World Health Organization’s self-care guidelines ^38^. Future strategies should further focus on patient empowerment and support to take an active role in the care and management of their conditions. These self-care approaches are especially important to supplement the limited care capacity, especially in human resources and medicines and supplies as has been noted for HIV care and other conditions ^39, 40^.

Whereas some of the patients’ coping mechanisms were important in helping them deal with the disease, others were concerning. For instance, some patients reportedly shared medication, others resorted to using conventional medicine together with herbal alternatives or skipped their medications. Sustained and tight glycaemic control and blood pressure control has been reported to optimize patient’s protection against cardiovascular outcomes ^41^, whereas discontinuation of treatment could be fatal. These practices may therefore turn out to be harmful to the patients and overall NCD control strategies. Adherence to recommended lifestyles is always challenging among NCD patients ^42, 43^. Our participants endeavoured to incorporate lifestyle recommendations into their daily routines, although some reported that some of the recommendations were unrealistic. A study in Eastern Uganda ^42^ found that patients did not show concern towards recommended lifestyles, although they perceived diabetes as a very severe disease. Thus, interventions to assist hypertension and diabetes patients to adopt recommended lifestyles are warranted. With NCDs being a relatively new phenomenon in SSA, gaps in capacity of health workers have been reported ^12– 14, 44^ and there is need to build their capacity in contextualising lifestyle recommendations for patients and equipping them with key information to manage their conditions. Broadly, by addressing health system challenges and putting in place proper patient support systems such as for self-management, follow-up and referral, better patient outcomes can be achieved ^45^. Efforts are also necessary to activate self-care systems beyond the patient such as families, peers, and other social support networks, and strengthening community health and primary health care systems to offer appropriate NCD services. Findings from this study informed the interventions designed by the SPICES project to better address the health system challenges in accessing care for cardiovascular disease. The interventions include training of health workers in CVD prevention, care, and management, instituting a regular screening process for adults at health facilities to ease diagnosis of the conditions and providing basic equipment to support the process. The SPICES project also introduced electronic data capture at the health facilities to improve CVD data and support follow-up of patients ^19^. Other activities of the SPICES project have been targeted at communities to support prevention of CVDs and early identification of at-risk individuals and empower them to seek health care early ^19^.

### Study strengths and limitations

This study was part of the situational analysis for the SPICES project and thus some issues especially around the facility-organised coping mechanisms were not explored in depth. We also had both male and female patients in the same FGD groups that were moderated by males and could have limited the expression of females. However, efforts were taken by the moderator to ensure involvement of all participants in the discussion and avoid any dominance by others including by directing questions to different participants and encouraging everyone to share their reflections. The patients also, anticipating the potential benefits of the study, were eager to participate and contribute. This study did not involve patients who primarily obtained services from private facilities and focussed on those from government and private not-for-profit facilities who were expected to be targeted by interventions. The patients were also at a higher health facility level (health centre IV and hospital) because most lower health facilities either did not provide or had less structured chronic care services. We also did not do participant checking of the data. Nevertheless, this study adds to the literature of patient experiences and their coping mechanisms in health systems in SSA countries.

## Conclusions

Chronic disease patients face several barriers in managing their conditions in unprepared health systems for non-communicable disease care and have thus adopted several measures to cope with their conditions, a reflection of self-care initiatives at both individual and community levels. These initiatives could be reinforced and supplemented with a functional community health and primary health care system to better support and empower patients as steps are taken to address existing challenges in care and management of chronic conditions.

## Data Availability

All data underlying the results cannot be made publicly available due to ethical restrictions related to protecting patient privacy and sufficient deidentification of transcripts and recordings. The transcripts can, however, be accessed through emailing the corresponding author and agreeing to ethical and access restrictions around data confidentiality. Figshare: Focus group discussion guide. https://doi.org/10.6084/m9.figshare.14213873.v1 ^23^. Figshare: COREQ checklist for “Hypertension and diabetes patients’ perspective of challenges and their coping mechanisms in Mukono and Buikwe districts in Uganda – a qualitative study”. http://10.6084/m9.figshare.14012246 ^25^. Data are available under the terms of the Creative Commons Attribution 4.0 International license (CC-BY 4.0).
