# Supplementary material for: Hypertension and diabetes patients’ perspective of challenges and their coping mechanisms in Mukono and Buikwe districts in Uganda – a qualitative study
Source: Open Res Eur. 2023 Sep 22;1:30. Originally published 2021 Mar 26. [Version 2] doi: 10.12688/openreseurope.13286.2 (PMC10831227; doi:10.12688/openreseurope.13286.2)
Supplement: Peer review history

# Reviewer report: Reviewer response for version 2

**DOI:** 10.21956/openreseurope.17988.r35241

**Published:** 2024-01-31

**Authors:** Anika L Hines

**Affiliations:** 1 Department of Health Behavior and Policy, Virginia Commonwealth University School of Medicine, Richmond, VA, USA

Hypertension and diabetes management remain core concerns of healthcare systems worldwide. Whereas health systems in the North American and European continents have traditionally focused on these non-communicable diseases, regions of Africa and Asia, have not developed the same capacity, as their primary focus has been infectious disease. This article is a qualitative inquiry of patient experiences in attaining care for hypertension and diabetes. Overall, the article is well written and the findings and points of discussion are appropriately contextualized within existing literature; however, there are some critical omissions from the methodological approach that detract from the scientific rigor of this qualitative work.

 The authors have adequately addressed my initial concerns regarding the documentation of methods in my initial review of this manuscript.

Is the study design appropriate and does the work have academic merit?

Partly

Is the work clearly and accurately presented and does it cite the current literature?

Yes

If applicable, is the statistical analysis and its interpretation appropriate?

Yes

Are all the source data underlying the results available to ensure full reproducibility?

No source data required

Are the conclusions drawn adequately supported by the results?

Yes

Are sufficient details of methods and analysis provided to allow replication by others?

No

Reviewer Expertise:

Hypertension; participatory research; social epidemiology; qualitative methods; social determinants of health; health disparities

I confirm that I have read this submission and believe that I have an appropriate level of expertise to confirm that it is of an acceptable scientific standard.

# Reviewer report: Reviewer response for version 2

**DOI:** 10.21956/openreseurope.17988.r35242

**Published:** 2023-10-13

**Authors:** Rose Clarke Nanyonga

**Affiliations:** 1 Institute of Public Health and Management, Clarke International University, Kampala, Uganda

The Authors have addressed all comments satisfactorily. I have no further comments at this time

Is the study design appropriate and does the work have academic merit?

Yes

Is the work clearly and accurately presented and does it cite the current literature?

Partly

If applicable, is the statistical analysis and its interpretation appropriate?

Not applicable

Are all the source data underlying the results available to ensure full reproducibility?

Partly

Are the conclusions drawn adequately supported by the results?

Yes

Are sufficient details of methods and analysis provided to allow replication by others?

Yes

Reviewer Expertise:

Non communicable diseases; Health systems research; Global Health.

I confirm that I have read this submission and believe that I have an appropriate level of expertise to confirm that it is of an acceptable scientific standard.

# Reviewer report: Reviewer response for version 1

**DOI:** 10.21956/openreseurope.14356.r29835

**Published:** 2022-08-31

**Authors:** Anika L Hines

**Affiliations:** 1 Department of Health Behavior and Policy, Virginia Commonwealth University School of Medicine, Richmond, VA, USA

Hypertension and diabetes management remain core concerns of healthcare systems worldwide. Whereas health systems on the North American and European continents have traditionally focused on these non-communicable diseases, regions of Africa and Asia, have not developed the same capacity, as their primary focus has been infectious disease. This article is a qualitative inquiry of patient experiences in attaining care for hypertension and diabetes. Overall, the article is well written and the findings and points of discussion are appropriately contextualized within existing literature; however, there are some critical omissions from the methodological approach that detract from the scientific rigor of this qualitative work. Please find enumerated points below:

1. Authors should describe the recruitment approach with more detail, including processes of how patients were recruited and by whom. 
2. Authors should also provide details on the sample population, including which patients were eligible to participate and exclusion criteria for the study. It would also be helpful to characterize demographics of participants (e.g., age range, sex or gender, income, occupation). Even in qualitative inquiry, the description of the population is critical to data interpretation, including limitations such as sample or selection bias. The authors have not established the sample population adequately.
3. There needs to be more information on the theoretical framework used to design the focus group discussion guide as well as that used to analyze the data. It seems that the investigators may have entered the inquiry with some ideas or guidance about what barriers individuals might encounter. Knowing what the participants were asked is critical in discerning the “weight” of their answers. For example, were the participants led to discuss certain challenges or did they surface organically? The authors also mention that there was a pre-tested focus group guide; however, it is unclear who were the subjects of this pretest. In addition, it is good practice to check with participants (member checking) to ensure that researchers' perceptions of the data adequately align with what study members aimed to convey as a validity check.

Is the study design appropriate and does the work have academic merit?

Partly

Is the work clearly and accurately presented and does it cite the current literature?

Yes

If applicable, is the statistical analysis and its interpretation appropriate?

Yes

Are all the source data underlying the results available to ensure full reproducibility?

No source data required

Are the conclusions drawn adequately supported by the results?

Yes

Are sufficient details of methods and analysis provided to allow replication by others?

No

Reviewer Expertise:

Hypertension; participatory research; social epidemiology; qualitative methods; social determinants of health; health disparities

I confirm that I have read this submission and believe that I have an appropriate level of expertise to confirm that it is of an acceptable scientific standard, however I have significant reservations, as outlined above.

# Response

**Published:** 2023-09-17

**Authors:** Rawlance Ndejjo

**Affiliations:** Makerere University, Uganda

We appreciate your review comments that has enabled us improve our manuscript. Here below are the responses to the raised comments.

**Comment 1:** **Authors should describe the recruitment approach with more detail, including processes of how patients were recruited and by whom.** 

 Response: The study participants were mobilized by the in-charge of the chronic care clinic ensuring a mix of male and female participants and age groups (young and older clients). The participants were invited to the clinic for the group discussions.

**Comment 2:** **Authors should also provide details on the sample population, including which patients were eligible to participate and exclusion criteria for the study. It would also be helpful to characterize demographics of participants (e.g., age range, sex or gender, income, occupation). Even in qualitative inquiry, the description of the population is critical to data interpretation, including limitations such as sample or selection bias. The authors have not established the sample population adequately.**

 Response: All hypertension and diabetes clients at the selected facilities were eligible to participate. These clients were mobilized by the health facility in charge considering their age and gender. Only participants who weren’t feeling well enough to participate in the discussion were excluded. We provided the characteristics of the study population as the first section of the results with information on age, sex, marital status, education level, occupation, chronic condition, and how long the participants had the condition which supports contextualizing our sample population.

**Comment 3:** **There needs to be more information on the theoretical framework used to design the focus group discussion guide as well as that used to analyze the data. It seems that the investigators may have entered the inquiry with some ideas or guidance about what barriers individuals might encounter. Knowing what the participants were asked is critical in discerning the “weight” of their answers. For example, were the participants led to discuss certain challenges or did they surface organically? The authors also mention that there was a pre-tested focus group guide; however, it is unclear who were the subjects of this pretest. In addition, it is good practice to check with participants (member checking) to ensure that researchers' perceptions of the data adequately align with what study members aimed to convey as a validity check.**

 Response: Our interview guide was informed by various literature that we highlight in the methods. We have also added detail on the analysis approach to clarify the process of codebook generation. Otherwise, the FGD guide that we attach was pretested among similar patients at two lower-level health facilities within the district. We did not do participant checking of the data and we highlight this as a limitation of the study.

# Reviewer report: Reviewer response for version 1

**DOI:** 10.21956/openreseurope.14356.r26657

**Published:** 2021-04-20

**Authors:** Rose Clarke Nanyonga

**Affiliations:** 1 Institute of Public Health and Management, Clarke International University, Kampala, Uganda

Thank you for the opportunity to review this work. I have a few comments that I hope will strengthen the manuscript.

 Abstract: Authors should consider reorganizing the results in the abstract to match the themes identified in the table and results section. Separating the themes into Challenge Themes and Coping Themes will enhance clarity for the reader.  At the moment the themes are presented as long long descriptive sentences in the abstract.

 Results

 In the table, authors present the following themes: Getting diagnosed with HTN and DM; Accessing care for HTN and DM; Accessing Medication, and Monitoring and Managing conditions. However in the text, the authors have an additional theme: adopting lifestyle recommendations?

 Consistence is needed in what is reflected in the abstract, results section and the results table.

 Discussion: Authors should strive to contexualise their results in existing literature and minimize repeating their findings in the discussion section. Some recommendations are made without any supporting data: e.g. last discussion paragraph ..."and there is need to build their capacity in contextualising lifestyle recommendations for patients and equipping them with key information to manage their conditions. Broadly, by addressing health system challenges and putting in place proper patient support systems such as for self-management, follow-up and referral and activating self-care systems beyond the patient such as families, peers and other social support networks, and strengthening community health and primary health care systems to offer appropriate NCD services, better patient outcomes can be achieved". Such important recommendations need to be supported by appropriate supporting literature to strengthen this section.

 Study strengths and Limitations

 Authors mention "This study was part of the situational analysis for the SPICES project and thus some issues were not explored in depth....such as? What other issues would have added value?

 Authors mention that efforts (such as?) were taken by the moderator to ensure involvement of all participants in the discussion and avoid any dominance by others.

 May be useful to mention in the methodology section as to why higher health facility levels were chosen.

Is the study design appropriate and does the work have academic merit?

Yes

Is the work clearly and accurately presented and does it cite the current literature?

Partly

If applicable, is the statistical analysis and its interpretation appropriate?

Not applicable

Are all the source data underlying the results available to ensure full reproducibility?

Partly

Are the conclusions drawn adequately supported by the results?

Yes

Are sufficient details of methods and analysis provided to allow replication by others?

Yes

Reviewer Expertise:

Non communicable diseases; Health systems research; Global Health.

I confirm that I have read this submission and believe that I have an appropriate level of expertise to confirm that it is of an acceptable scientific standard, however I have significant reservations, as outlined above.

# Response

**Published:** 2023-09-17

**Authors:** Rawlance Ndejjo

**Affiliations:** Makerere University, Uganda

Thank you for your review of our article that has enabled us improve it. Here below are the responses to the raised comments.

**Comment 1:** **Abstract: Authors should consider reorganizing the results in the abstract to match the themes identified in the table and results section. Separating the themes into Challenge Themes and Coping Themes will enhance clarity for the reader.  At the moment the themes are presented as long long descriptive sentences in the abstract.**

 Response: Thank you for this comment. As we had cross-cutting themes cutting across both barriers and facilitators, we believe presenting them together as is done within the results of the manuscript is more concise, representative and informative. In the write-up, we add context to indicate the barriers and facilitators. Moreover, given the word count, separating the subthemes into barriers and facilitators would require a larger word count.

**Comment 2:** **Results** **-** **In the table, authors present the following themes: Getting diagnosed with HTN and DM; Accessing care for HTN and DM; Accessing Medication, and Monitoring and Managing conditions. However in the text, the authors have an additional theme: adopting lifestyle recommendations?** **Consistence is needed in what is reflected in the abstract, results section and the results table.**

 Response: Our study had five themes which are consistently presented in the abstract, results, and also within the summary table. We have edited the first theme in the table from ‘ *Getting diagnosed with HTN and DM’* to ‘ *Diagnosis with hypertension and diabetes’* for consistency. Thank you.

**Comment 3:** **Discussion: Authors should strive to contexualise their results in existing literature and minimize repeating their findings in the discussion section. Some recommendations are made without any supporting data: e.g. last discussion paragraph ..."and there is need to build their capacity in contextualising lifestyle recommendations for patients and equipping them with key information to manage their conditions. Broadly, by addressing health system challenges and putting in place proper patient support systems such as for self-management, follow-up and referral and activating self-care systems beyond the patient such as families, peers and other social support networks, and strengthening community health and primary health care systems to offer appropriate NCD services, better patient outcomes can be achieved". Such important recommendations need to be supported by appropriate supporting literature to strengthen this section.**

 Response: We have now added additional contextual literature for the recommendations we make.  

**Comment 4:** **Study strengths and Limitations** **-** **Authors mention "This study was part of the situational analysis for the SPICES project and thus some issues were not explored in depth....such as? What other issues would have added value?**

 Response: The issues that we did not explore in depth include the facility-organised coping mechanisms and we have now highlighted this within the manuscript.

**Comment 5:** **Authors mention that efforts (such as?) were taken by the moderator to ensure involvement of all participants in the discussion and avoid any dominance by others.**

 Response: The moderator ensured that everyone in the group was given a chance to speak and encouraged to share their views. The moderator also directed some questions to different FGD participants before others shared their reflections.

**Comment 6:** **May be useful to mention in the methodology section as to why higher health facility levels were chosen.**

 Response: We have highlighted that higher-level facilities were selected because this is where care and follow-up for hypertension and diabetes were more structured and consistent with established clinics.
